# Safety and efficacy of kidney transplantation in patients with aortoiliac stenosis: a retrospective cohort study

**DOI:** 10.1097/JS9.0000000000000926

**Published:** 2023-11-27

**Authors:** Yitian Fang, Julie J.M. Hamm, Floris P.J. den Hartog, Hendrikus J.A.N. Kimenai, Ron W.F. de Bruin, Robert C. Minnee

**Affiliations:** a Erasmus MC Transplant Institute, Department of Surgery, Division of HPB and Transplant Surgery; b Department of Surgery, Erasmus Medical Center, Rotterdam, The Netherlands

**Keywords:** artery stenoses, graft survival, iliac artery, kidney transplantation, patient survival, peripheral vascular disease

## Abstract

**Background:**

The impact of aortoiliac occlusive disease on kidney transplantation remains unclear. This study aims to investigate the clinical outcomes of kidney transplant patients with aortoiliac atherosclerotic stenosis.

**Methods:**

Retrospective data from our transplant center were used to identify patients undergoing kidney transplantation between January 2010 and December 2020. Aortoiliac atherosclerotic stenosis was screened and stratified by the Trans-Atlantic Inter-Society Consensus (TASC) II classification. The primary outcome was patient survival. Secondary outcomes were 90-day mortality, death-censored graft survival, graft function, and arterial complications. Propensity score matching was used to match all patients in the stenosis group with patients without stenosis sharing similar characteristics.

**Results:**

The analysis included 655 patients, 524 without stenosis and 131 with aortoiliac stenosis (95 with TASC A/B stenosis and 36 with TASC C/D stenosis). Recipient age [median (IQR), 66 (60–70) vs. 66 (59–71) years; *P*=0.47], sex [male: 87 (66%) vs. 355 (68%), *P*=0.85], and comorbidities were comparable between the stenosis and no-stenosis groups. Forty-six (35%) patients with stenosis were symptomatic. Patient survival was significantly lower in the stenosis group compared with the no-stenosis group (TASC A/B: 30.6% vs. no-stenosis: 44.1%, *P*=0.013; TASC C/D: 11.4% vs. no-stenosis: 44.1%, *P*<0.001). The incidence rates of artery dissection, lower extremity ischemia, and acute thrombosis were significantly higher in the stenosis group (*P*<0.001). However, death-censored graft survival (TASC A/B: 73.6% vs. no-stenosis: 72.9%, *P*=0.62; TASC C/D: 58.1% vs. no-stenosis: 72.9%, *P*=0.16) and graft function were comparable between the groups.

**Conclusions:**

Aortoiliac atherosclerotic stenosis significantly impacts patient survival but not graft survival. Our analyses suggest that patients with TASC A/B stenosis have prolonged survival and enhanced quality of life through kidney transplantation. However, for patients with TASC C/D stenosis, kidney transplantation improves quality of life without bringing survival benefits.

## Introduction

Highlights

- The long-term patient survival was significantly lower in patients with aortoiliac stenosis compared with those without stenosis.
- Aortoiliac stenosis has minimal impact on death-censored graft survival and graft function.
- Aortoiliac stenosis should not be a contraindication of kidney transplantation for patients with TASC A/B stenosis.

Kidney transplantation is the optimal treatment for end-stage renal disease (ESRD)^1^. Unfortunately, not all ESRD patients are eligible for kidney transplantation. Aortoiliac atherosclerotic stenosis, a common type of peripheral artery occlusive disease in ESRD patients, is considered a relative contraindication for kidney transplantation^2^. Previous studies suggest that ESRD patients are prone to atherogenesis and aortoiliac atherosclerotic stenosis, likely due to uremia-associated inflammation, high-density lipoprotein (HDL) dysfunction, and uremic vasculopathy^3^. Increased numbers of specific proinflammatory subsets of T cells and monocytes contribute to the destabilization of atherosclerotic plaques^4,5^. HDL from ESRD patients is rendered a damaging particle that promotes endothelial dysfunction, vasoconstriction, and inflammatory activation^6^. Additionally, the hypertrophy, proliferation, and calcification of vascular smooth muscle cells have been reported to play a pivotal role in uremic vasculopathy^7^. Kidney transplantation in this population has raised several concerns, including the complexity of vascular anastomosis and inferior life expectancy^8–11^. Consequently, it is still debated whether patients with aortoiliac atherosclerotic stenosis would benefit from kidney transplantation.

Aortoiliac stenosis can be classified according to the Trans-Atlantic Inter-Society Consensus (TASC) II classification^12^. TASC A and B stenosis refer to mild or moderate narrowing of the aortoiliac artery, while TASC C and D stenosis are severe stenosis or occlusion which can extend into the femoral artery. For TASC A and B stenosis, endovascular or endoluminal interventions are recommended, whereas TASC C and D stenosis requires surgical management as the first approach^13,14^. Several studies have reported inferior survival outcomes in kidney transplant patients with TASC C/D stenosis^15,16^. However, these studies exclusively involved patients who underwent contrast-enhanced imaging. Although contrast-enhanced imaging provides a more detailed visualization of the stenosis compared with vascular ultrasound, the contrast agents used can be nephrotoxic. Therefore, it is not a standard pretransplant screening procedure, leading to a selection bias in the research subjects. Moreover, the baseline characteristics of patients with and without stenosis in the studies were not comparable, undermining the robustness of their research conclusions.

This study aimed to investigate the clinical outcomes of kidney transplantation in patients with aortoiliac atherosclerotic stenosis. The patient survival, graft survival, graft function, and complications were systematically compared. We enrolled low-risk patients and used propensity score matching (PSM) to minimize selection bias and confounding factors.

## Methods

### Study design and data collection

We conducted a retrospective, single-center, observational study using our transplant database. This study was approved by the Medical Ethics Committee and registered on ClinicalTrials.gov. This work has been reported in line with the STROCSS criteria (Supplemental Digital Content 1, http://links.lww.com/JS9/B454)^17^.

We identified all patients undergoing kidney transplantation in our center between January 2010 and December 2020. The exclusion criteria were age less than 18 years when transplanted, combined organ transplantation, or no follow-up data. These patients were categorized into the no-stenosis group and the stenosis group based on the presence of aortoiliac stenosis. To minimize potential confounding factors between the two groups, we used PSM to match patients in the stenosis group with individuals from the no-stenosis group who shared similar characteristics.

As a standard pretransplant assessment, all patients underwent artery duplex sonography examination of aortoiliac vessels, which has proven to be reliable for the detection of aortoiliac stenosis^18^. When stenosis was detected, contrast-enhanced computed tomography (CT) or MRI of the aortoiliac segment was used to obtain anatomical information and assess the severity. Aortoiliac stenosis was defined as lumen narrowing greater than 50% in the aortoiliac artery. Patients were regularly followed-up by our nephrologists, with all relevant data recorded in the electronic patient record system. The follow-up duration extended until the patient’s death or January 2023. During this time frame, we compared the clinical outcomes, such as patient survival, graft function, and arterial complications between the groups.

### Outcome measurements

The primary outcome was patient survival. The secondary outcomes were 90-day mortality, death-censored graft survival, graft function, and arterial complications. To estimate renal function, the estimated glomerular filtration rate (eGFR) was calculated using the Chronic Kidney Disease Epidemiology Collaboration (CKD-EPI) equation at 1, 3, and 6 months and each posttransplant year^19^. Follow-up assessment was performed using a follow-up index (FUI)^20^.

Patient survival and 90-day mortality were calculated from the date of transplantation until the endpoints. Death-censored graft survival was defined as the time from transplant to graft failure, with censoring for death with a functioning graft. All arterial complications relevant to kidney transplantation were reported. FUI was calculated as the ratio between the actual investigation period and the potential follow-up period. A FUI value closer to 1 indicates a smaller risk of selection bias^20^.

### Statistical analysis

Continuous variables were presented as medians with interquartile ranges (IQRs). Categorical variables were presented as frequencies and percentages. PSM was performed in a nearest neighbor-matching approach with a maximum caliper of 0.1 in a 1 : 4 ratio^21^. The matching parameters included age, dialysis mode, retransplant, donor type, comorbidities, and smoking status. The Mann–Whitney *U* test was used to compare continuous variables, and the *χ*^2^ test was used to compare categorical variables. Kaplan–Meier curves and log-rank tests were used to compare patient and graft survival. A *P* value of pairwise multiple comparisons was adjusted with the Benjamini–Hochberg procedure. The relative risk of aortoiliac stenosis on patient mortality and graft failure was assessed using the Cox regression model. A two-sided *P*<0.05 was considered statistically significant. All analyses were conducted using R version 4.2.2.

## Results

### Study cohort

**Figure 1:**
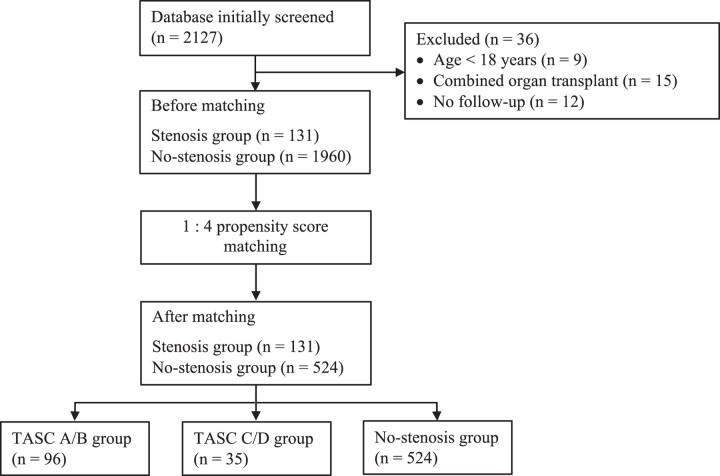
Flow diagram of study screening, recruitment, and enrollment. TASC, Trans-Atlantic Inter-Society Consensus.

We initially identified 2127 patients who had undergone kidney transplantation in our center between January 2010 and December 2020 (Fig. 1). Thirty-six patients were excluded due to age under 18 years at transplant (*n*=9), combined organ transplantation (*n*=15), or no follow-up data (*n*=12). Among the remaining patients, 131 patients were diagnosed with pretransplant stenosis, and 1960 patients without stenosis. These patients were enrolled in PSM, yielding a final cohort of 131 patients in the stenosis group and 524 matched patients in the no-stenosis group. In the stenosis group, 96 patients were classified with TASC A/B stenosis and 35 with TASC C/D stenosis according to the TASC II classification.

### Baseline characteristics

Before PSM, patients in the stenosis group were significantly older at transplantation than patients in the no-stenosis group [median (IQR), 66 (60–70) vs. 58 (47–66) years; *P*<0.001]. The proportion of male gender was similar between the two groups [87 (66%) vs. 1214 (62%), *P*=0.35]. More patients in the stenosis group underwent dialysis compared with the patients in the no-stenosis group [102 (78%) vs. 1294 (66%), *P*=0.021]. The duration of dialysis was comparable. There was a significantly higher incidence of diabetes mellitus [72 (55%) vs. 540 (28%), *P*<0.001], cardio-cerebrovascular disease [68 (52%) vs. 466 (24%), *P*<0.001] and smoking [93 (71%) vs. 870 (44%), *P*<0.001] in the stenosis group.

After PSM, recipient age [median (IQR), 66 (60–70) vs. 66 (59–71) years; *P*=0.47], sex [male: 87 (66%) vs. 355 (68%), *P*=0.85], diabetes mellitus [72 (55%) vs. 284 (54%), *P*=0.95], hypertension [92 (70%) vs. 366 (70%), *P*=0.99], cardio-cerebrovascular disease [68 (52%) vs. 251 (48%), *P*=0.47], and other demographics were comparable between the stenosis and no-stenosis groups. There was no statistical difference in the distribution of ESRD causes [vascular: 37 (28%) vs. 160 (31%); diabetes mellitus: 46 (35%) vs. 152 (29%); glomerulonephritis: 23 (18%) vs. 76 (15%); polycystic kidney: 7 (5%) vs. 48 (9%); interstitial disease: 5 (4%) vs. 21 (4%); others/unknown: 13 (10%) vs. 66 (13%); *P*=0.57] between the two groups. The median follow-up was 4.8 years (IQR, 3.0–7.2), corresponding to a mean FUI of 0.98 ± 0.10 relative to the predefined end date. No patients had been lost to follow-up within 30 days after kidney transplantation, and 279 deaths (42.6%) were known to have occurred after a median follow-up of 4.1 years (IQR, 1.9–6.2; FUI=1). Twenty-four patients were ‘potential survivors’ with an FUI less than 1 (Table 1).

**Table 1:** Baseline characteristics before and after propensity score matching.

	Before matching			After matching		
	No-stenosis (*n*=1960)	Stenosis (*n*=131)	*P*	No-stenosis (*n*=524)	Stenosis (*n*=131)	*P*
Age (years)	58 (47–66)	66 (60–70)	<0.001	66 (59–71)	66 (60–70)	0.47
Male sex	1214 (62)	87 (66)	0.35	355 (68)	87 (66)	0.85
BMI (kg/m^2^)	26 (23–30)	28 (24–32)	0.092	28 (24–31)	28 (24–32)	0.71
Cause of ESRD			<0.001			0.57
Vascular	511 (26)	37 (28)		160 (31)	37 (28)	
Diabetes mellitus	303 (16)	46 (35)		152 (29)	46 (35)	
Glomerulonephritis	487 (25)	23 (18)		76 (15)	23 (18)	
Polycystic kidney disease	242 (12)	7 (5)		48 (9)	7 (5)	
Interstitial disease	86 (4)	5 (4)		21 (4)	5 (4)	
Others/unknown	331 (17)	13 (10)		66 (13)	13 (10)	
Type of dialysis			0.021			0.98
Pre-emptive	666 (34)	29 (22)		120 (23)	29 (22)	
Hemodialysis	860 (44)	74 (57)		297 (57)	74 (57)	
Peritoneal dialysis	278 (14)	17 (13)		69 (13)	17 (13)	
Combined modality	156 (8)	11 (8)		38 (7)	11 (8)	
Duration of dialysis (months)	22 (12–38)	23 (15–38)	0.27	22 (11–36)	23 (15–38)	0.20
Retransplant	329 (17)	21 (16)	0.92	87 (17)	21 (16)	0.98
Donor type			0.090			0.85
Living donor	1209 (62)	70 (53)		290 (55)	70 (53)	
DBD	310 (16)	21 (16)		87 (17)	21 (16)	
DCD	441 (23)	40 (31)		147 (28)	40 (31)	
Comorbidities						
Diabetes mellitus	540 (28)	72 (55)	<0.001	284 (54)	72 (55)	0.95
Hypertension	1239 (63)	92 (70)	0.13	366 (70)	92 (70)	0.99
CCVD	466 (24)	68 (52)	<0.001	251 (48)	68 (52)	0.47
Smoking status			<0.001			0.99
Never smoker	1090 (56)	38 (29)		150 (29)	38 (29)	
Former smoker	570 (29)	65 (50)		261 (50)	65 (50)	
Current smoker	300 (15)	28 (21)		113 (22)	28 (21)	

Data are presented as *n* (%) or median (interquartile range).

BMI, body mass index; CCVD, cardio-cerebrovascular disease; DBD, donation after brain death; DCD, donation after circulatory death; ESRD, end-stage renal disease.

### Pretransplant symptoms and interventions

Among the patients with aortoiliac atherosclerotic stenosis, 46 (35%) patients presented symptoms, including claudication, lower limb pain, and limb gangrene (28 with TASC A/B stenosis and 18 with TASC C/D stenosis). For these patients, a comprehensive multidisciplinary meeting involving transplant surgeons, vascular surgeons, and nephrologists was organized to determine the pretransplant intervention. A conservative approach was adopted for 15 patients. Percutaneous transluminal angioplasty and stent placement were performed in 24 patients. Femoral popliteal bypass surgery was performed on seven patients. Peritransplant endarterectomy was performed in three patients. All 10 patients ultimately required amputation. Additionally, 13 symptomatic patients and 30 asymptomatic patients suffered from coronary occlusive disease.

### Patient survival

**Figure 2:**
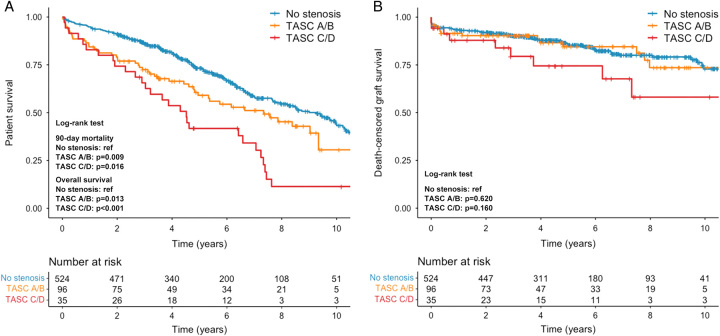
Kaplan–Meier curves comparing (A) patient survival and (B) death-censored graft survival in transplant patients stratified by TASC classification. *P* values of pairwise multiple comparisons were adjusted with the Benjamini–Hochberg procedure. TASC, Trans-Atlantic Inter-Society Consensus.

The differences in survival rates are shown in Figure 2A. Compared with the no-stenosis group, a significantly higher 90-day mortality was observed in patients with aortoiliac stenosis (no-stenosis vs. TASC A/B stenosis: 1.9% vs. 7.3%, *P*=0.009; no-stenosis vs. TASC C/D stenosis: 1.9% vs. 8.6%, *P*=0.016). Among patients who died within 90 posttransplant days in the stenosis group (7 in TASC A/B group and 3 in TASC C/D group), the primary causes of death were cardiovascular events (*n*=4), sepsis (*n*=4), and respiratory failure (*n*=2).

By the end of the follow-up, there were 202 (39%) deaths in the no-stenosis group, 48 (50%) in the TASC A/B group, and 29 (83%) in the TASC C/D group. Kaplan–Meier survival analysis revealed a significant difference in overall survival between the no-stenosis group and TASC A/B group (*P*=0.013), as well as the no-stenosis group and TASC C/D group (*P*<0.001). The survival rates at 1, 3, 5, and 10 years in the TASC A/B group (84.4%, 72.5%, 59.1% and 30.6%, respectively) and TASC C/D group (82.9%, 65.6%, 41.7% and 11.4%, respectively) were significantly worse than those in the no-stenosis group (94.8%, 86.1%, 73.4%, and 44.1%, respectively). Cox regression analysis showed an increased risk of patient mortality associated with both TASC A/B stenosis (HR 1.49, 95% CI 1.09–2.04, *P*=0.013) and TASC C/D stenosis (HR 2.66, 95% CI 1.80–3.93, *P*<0.001; Table 2).

**Table 2:** Hazard ratios of aortoiliac stenosis for patient mortality and graft failure.

	Patient mortality			Death-censored graft failure		
	HR	95% CI	*P*	HR	95% CI	*P*
TASC A/B stenosis	1.49	1.09–2.04	0.013	1.15	0.67–1.96	0.622
TASC C/D stenosis	2.66	1.80–3.93	<0.001	1.94	0.98–3.87	0.059

CI, confidence interval; HR, hazard ratio; TASC, Trans-Atlantic Inter-Society Consensus.

### Graft survival

In total, 106 cases of graft failure occurred, with 81 (15%) in the no-stenosis group, 16 (17%) in the TASC A/B group, and 9 (26%) in the TASC C/D group. The death-censored graft survival rates were similar between the no-stenosis group and the TASC A/B group (*P*=0.620). At 1, 3, 5, and 10 years, the death-censored graft survival rates were 93.5%, 90.0%, 85.8%, and 72.9%, respectively, for the no-stenosis group, and 91.5%, 90.3%, 84.5%, and 73.6%, respectively for the TASC A/B group. Patients in the TASC C/D group had inferior graft survival rates at 1, 3, 5, and 10 years (87.9%, 79.5%, 74.5%, and 58.1%, respectively) compared with those in the no-stenosis group, but the difference was not significant (*P*=0.160) (Fig. 2B). The survival curves align with the Cox regression analysis, showing that both TASC A/B stenosis and TASC C/D stenosis are not risk factors for graft failure (TASC A/B: HR 1.15, 95% CI 0.67–1.96, *P*=0.622; TASC C/D: HR 1.94, 95% CI 0.98–3.87, *P*=0.059; Table 2).

### Graft function

**Figure 3:**
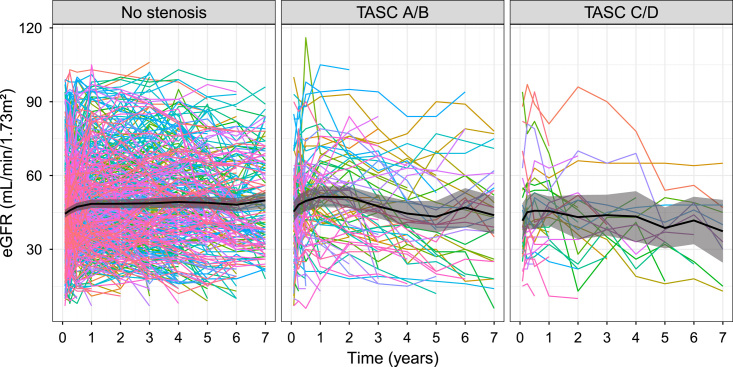
Spaghetti plots of renal function stratified by aortoiliac stenosis classification. eGFR, estimated graft filtration rate; TASC, Trans-Atlantic Inter-Society Consensus.

The spaghetti plots of eGFR stratified by TASC classification are shown in Figure 3. Patients in the no-stenosis group displayed stable graft function during the long-term follow-up, whereas a fluctuation and decline were observed in both TASC A/B and TASC C/D groups. However, there was no significant difference in eGFR among patients in the TASC A/B and TASC C/D groups compared with those in the no-stenosis group each posttransplant year (Supplemental Table 1, Supplemental Digital Content 2, http://links.lww.com/JS9/B455 and Supplemental Table 2, Supplemental Digital Content 2, http://links.lww.com/JS9/B455).

### Arterial complications and management

In the TASC A/B group, arterial complications occurred in four (4%) patients. Three patients had intraoperative external iliac artery dissection and underwent arterial reconstruction, with good recovery of renal function. Another patient had acute lower extremity ischemia caused by intraoperative clamping of the external iliac artery, and symptoms improved after stenting and angioplasty. In the TASC C/D group, three (9%) patients encountered arterial complications. One patient had acute arterial thrombosis and underwent heparin anticoagulation and thrombectomy, successfully restoring graft function. Another patient had artery kinking and renal perfusion improved after reanastomosis. Unfortunately, one patient had an artery prosthesis rupture, followed by failed reanastomosis and transplantectomy. In the matched no-stenosis group, six (1%) patients experienced arterial complications, including artery kinking, artery dissection, arterial thrombosis, anastomotic bleeding, and anastomotic stenosis. Five of these patients preserved graft function after reanastomosis or reoperation, while one patient required transplantectomy. The incidence of arterial complications was significantly higher among patients with aortoiliac stenosis (*P*<0.001).

### Subgroup analysis based on graft placement

**Figure 4:**
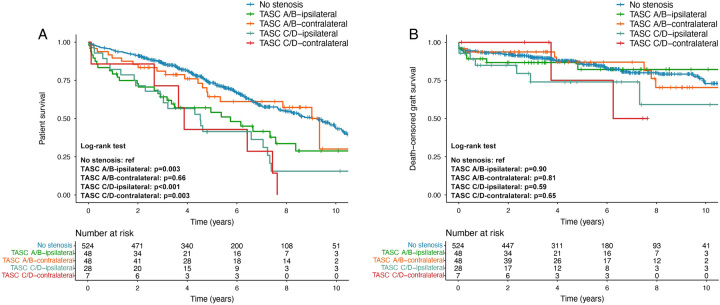
Kaplan–Meier curves comparing (A) patient survival and (B) death-censored graft survival in transplant recipients stratified by TASC classification and graft placement. *P* values of pairwise multiple comparisons were adjusted with the Benjamini–Hochberg procedure. TASC, Trans-Atlantic Inter-Society Consensus.

Subgroup survival analyses based on graft placement are presented in Figure 4. In the TASC A/B group, 48 patients underwent ipsilateral graft placement, while the other 48 received contralateral graft placement. Patients undergoing ipsilateral graft placement showed 1-year, 3-year, and 5-year survival rates of 79.2%, 64.2%, and 54.0%, respectively, which was significantly lower than the no-stenosis group (*P*=0.003). In contrast, for those undergoing contralateral graft placement, the corresponding rates were 89.6%, 81.0%, and 64.3%, demonstrating no significant difference compared with the no-stenosis group (*P*=0.66). In the TASC C/D group, 28 patients received the grafts on the ipsilateral side, while seven patients received grafts on the contralateral side. The 1-year, 3-year, and 5-year survival rates were 82.1%, 64.1%, and 41.5% for ipsilateral graft placement and 85.7%, 71.4%, and 42.9% for contralateral graft placement, respectively. Both rates were significantly lower than the no-stenosis group (*P*<0.001 and *P*=0.003, Fig. 4A).

For the death-censored graft survival, patients in the TASC A/B group who underwent ipsilateral graft placement displayed death-censored graft survival rates of 89.2%, 86.8%, and 82.2% at 1, 3, and 5 years, and patients with contralateral graft placement displayed rates of 93.7%, 93.7%, and 87.0%, respectively. In the TASC C/D group, patients who underwent ipsilateral graft placement displayed death-censored graft survival rates of 84.9%, 74.0%, and 74.0%, while those with contralateral graft placement displayed rates of 100%, 100%, and 75.0%, respectively. All subgroups achieved comparable graft survival as the no-stenosis group (Fig. 4B) (Table 3).

**Table 3:** Risk factor analysis for graft failure in transplant patients with aortoiliac stenosis.

	Univariate analysis			Multivariate analysis		
	HR	95% CI	*P*	HR	95% CI	*P*
Male sex	1.52	0.60–3.81	0.38			
Age ≥65 years	1.25	0.56–2.78	0.59			
BMI ≥30 kg/m^2^	1.76	0.78–3.95	0.17			
Dialysis type						
Pre-emptive	1	Reference				
Hemodialysis	1.12	0.43–2.89	0.82			
Peritoneal dialysis	0.96	0.24–3.85	0.95			
Combined modality	0.52	0.06–4.31	0.54			
Retransplant	2.26	0.89–5.73	0.085	1.87	0.72–4.85	0.20
Comorbidities						
CCVD	0.96	0.44–2.12	0.93			
Hypertension	0.62	0.28–1.37	0.24			
Diabetes mellitus	0.74	0.34–1.62	0.45			
Smoking history	1.22	0.49–3.07	0.67			
Deceased donor	3.33	1.42–7.83	0.006	2.84	1.18–6.83	0.020
TASC C/D stenosis	1.86	0.82–4.23	0.14	1.63	0.71–3.78	0.25

BMI, body mass index; CCVD, cardio-cerebrovascular disease; CI, confidence interval; HR, hazard ratio; PVD, peripheral vascular disease; TASC, Trans-Atlantic Inter-Society Consensus.

## Discussion

In this retrospective cohort study, we utilized a large transplant database to investigate the clinical outcomes of kidney transplantation in patients with aortoiliac atherosclerotic stenosis, assessing whether aortoiliac atherosclerotic stenosis should be a contraindication for kidney transplantation. By providing these insights, we hope this study can support clinical decision-making for ESRD patients with aortoiliac stenosis.

The patients in the stenosis group exhibited older age, a higher prevalence of diabetes mellitus, smoking, and dialysis. These factors play a crucial role in the development of peripheral arterial disease, including aortoiliac atherosclerotic stenosis^22–26^. Based on the fact that cardiovascular events are the leading cause of early posttransplant mortality, our data indicates that aortoiliac stenosis augments vascular disease burden and further increases the risk of 90-day mortality^27,28^. This finding is in line with the study by Babakry *et al*.^16^, which reported that patients with aortoiliac stenosis had significantly inferior cardiovascular event-free survival.

Furthermore, we investigated the impact of aortoiliac atherosclerotic stenosis on long-term patient outcomes. Our data demonstrate that the 5-year survival rates of patients in the TASC A/B group and TASC C/D group were 59.1% and 41.7%, respectively. In comparison to the survival data of ESRD patients subjected to dialysis, which remains ~40%, patients with TASC A/B stenosis can derive substantial survival benefits from kidney transplantation^29,30^. Nevertheless, the survival benefits appear marginal for patients with TASC C/D stenosis, as the survival gap is not significant.

The eligibility of ESRD patients with aortoiliac stenosis for transplantation remains controversial. Contraindications on life expectancy are variable and may differ depending on the institutional and geographic contexts. According to the European Society for Organ Transplantation (ESOT), a life expectancy of less than 2 years is an absolute contraindication for kidney transplantation^31^. However, the American Society of Transplantation (AST) recommends a minimum life expectancy of 5 years to be considered for kidney transplantation^32^. The study by Rijkse *et al*.^15^ showed that it is safe to perform kidney transplantation in patients with TASC A/B stenosis, while patients with TASC C/D stenosis have a significantly higher mortality, making kidney transplantation inadvisable. Our data reveals that patients with TASC A/B stenosis align with eligibility standards under both guidelines, evidenced by 2-year and 5-year survival rates of 79.1% and 59.1%, respectively. For patients with TASC C/D stenosis, the scenario becomes nuanced. This high-risk cohort appears to meet the ESOT criterion, with a 2-year survival rate of 74.3%, yet the AST guideline presents a potential mismatch, as the 5-year survival rate stands at 41.7%.

We also looked into the impact on long-term graft outcomes. Surprisingly, the presence of stenosis did not significantly impact death-censored graft survival. Similar cases were reported by Voiculescu *et al*.^33^ showing that in patients with proximal transplant renal artery stenosis, vascular problems could be managed without causing graft loss. Additionally, as a valid surrogate of renal function, posttransplant eGFR revealed a comparable performance between patients with TASC A/B or C/D stenosis and those without stenosis. This finding underscores the resilience of grafts against aortoiliac stenosis, highlighting the possibility of achieving satisfactory graft survival and function across varying severities of stenosis, thereby bringing an enhanced quality of life to these patients.

In terms of graft placement, a considerably higher proportion of ipsilateral kidney implantation was observed in the TASC C/D group compared and the TASC A/B group, likely due to the higher incidence of bilateral stenosis in the TASC C/D group. Among patients who underwent ipsilateral kidney implantation, more patients with TASC A/B stenosis underwent endovascular intervention or no intervention. In contrast, those with TASC C/D stenosis were more likely to undergo surgical intervention, reflecting the varying treatment strategies depending on the severity of the stenosis. Both endovascular and surgical interventions have been demonstrated to be safe and effective in the treatment of arterial occlusive disease with kidney transplantation^13,14,34,35^. Our data indicate that patients with TASC A/B stenosis who received contralateral graft placements showed significantly better long-term survival, while graft survival was comparable between different graft implantation sites. However, due to the limited subgroup size, more studies with larger populations are needed to validate this finding.

The basic principle of kidney transplantation is that the patient should derive overall benefit from transplantation compared with the alternative of dialysis. For patients with TASC A/B stenosis, kidney transplantation effectively prolongs survival and improves the quality of life. However, for those with TASC C/D stenosis, kidney transplantation distinctly improves their quality of life but cannot provide corresponding survival benefits. This paradox heralds an additional layer of complexity in the decision-making process for kidney allocation. The intricate balance of potentially extending the life expectancy of an individual through kidney transplantation while weighing against potentially better long-term survival outcomes for other recipients introduces a formidable challenge that necessitates careful consideration.

Our study has some limitations to be addressed. Firstly, as a retrospective study, there is a guarantee for selection bias in patients with the most severe aortoiliac stenosis, as these patients might be rejected for kidney transplantation and would not be included in the study. Secondly, the study’s single-center design could introduce institutional-specific practices that may not be universally applicable. Variations in surgical techniques and postoperative management could influence outcomes and limit generalizability. Multicenter studies with larger group sizes are needed to reach safe conclusions. Lastly, details of stenosis proximal or distal to the transplanted kidney were unknown, which could impact graft survival and function.

## Conclusion

Aortoiliac atherosclerotic stenosis significantly deteriorates patient survival, but not graft survival of kidney transplant patients. Nevertheless, patients with TASC A/B stenosis can experience prolonged survival and an enhanced quality of life through kidney transplantation, thus challenging the notion of aortoiliac stenosis as a contraindication. In contrast, while kidney transplantation can substantially improve the quality of life for patients with TASC C/D stenosis, it might not bring corresponding survival benefits. The decision regarding kidney transplantation for these individuals necessitates careful consideration. Extensive pretransplant, peritransplant, and posttransplant cardiovascular risk management is required in this high-risk population.

## Ethical approval

The study was approved by the Medical Ethics Committee of the Erasmus Medical Center (MEC-2017-1039).

## Consent

The requirement for informed consent was not applicable according to the local ethics committee because this was a noninterventional study and a high proportion of the population had passed away due to the long observation time.

## Source of funding

None.

## Author contribution

Y.F. and J.J.M.H.: participated in research design, data collection, data analysis, and paper writing; F.P.J.dH.: participated in data analysis and critical revision; H.J.A.N.K., R.W.F.dB., and R.C.M.: participated in research design and critical revision.

## Conflicts of interest disclosure

The authors declare no conflicts of interest.

## Research registration unique identifying number (UIN)

1. Name of the registry: ClinicalTrials.gov.
2. Unique identifying number or registration ID: NCT06020534.
3. Hyperlink to your specific registration (must be publicly accessible and will be checked): https://clinicaltrials.gov/ct2/show/NCT06020534

## Guarantor

R.C. Minnee.

## Data availability statement

Data sharing are not applicable to this article.

## Provenance and peer review

Not commissioned, externally peer-reviewed.

## Supplementary Material
