# Supplementary material for: Safety and efficacy of kidney transplantation in patients with aortoiliac stenosis: a retrospective cohort study
Source: Int J Surg. 2023 Nov 27;110(2):992–9. doi: 10.1097/JS9.0000000000000926 (PMC10871560; doi:10.1097/JS9.0000000000000926)
Supplement: Supplementary file 2 [file js9-110-0992-s002.docx]

**Supplemental Table 1.** Comparison of graft function between matched no-stenosis and TASC A/B patients

|  | eGFR (mL/min/1.73m^2^) | | *p* value |
| --- | --- | --- | --- |
|  | No stenosis group | TASC A/B group |  |
| 1 month | 43 (33-54) | 47 (33-58) | 0.35 |
| 3 months | 44 (34-55) | 48 (35-59) | 0.46 |
| 6 months | 44 (35-58) | 47 (36-58) | 0.26 |
| 1 year | 46 (35-59) | 49 (39-61) | 0.23 |
| 2 years | 46 (35-60) | 49 (36-62) | 0.22 |
| 3 years | 47 (34-61) | 46 (35-60) | 0.77 |
| 4 years | 48 (34-63) | 44 (33-58) | 0.38 |
| 5 years | 48 (36-61) | 43 (32-59) | 0.10 |
| 6 years | 47 (35-63) | 46 (32-60) | 0.70 |
| 7 years | 47 (34-65) | 47 (31-56) | 0.33 |

Data are presented as median (interquartile range). eGFR, estimated glomerular filtration rate.

**Supplemental Table 2.** Comparison of graft function between matched no-stenosis and TASC C/D patients

|  | eGFR (mL/min/1.73m^2^) | | *p* value |
| --- | --- | --- | --- |
|  | No stenosis group | TASC C/D group |  |
| 1 month | 43 (33-54) | 38 (28-53) | 0.24 |
| 3 months | 44 (34-55) | 40 (34-55) | 0.57 |
| 6 months | 44 (35-58) | 39 (32-56) | 0.44 |
| 1 year | 46 (35-59) | 46 (34-54) | 0.62 |
| 2 years | 46 (35-60) | 42 (27-52) | 0.22 |
| 3 years | 47 (34-61) | 40 (35-47) | 0.56 |
| 4 years | 48 (34-63) | 42 (28-55) | 0.38 |
| 5 years | 48 (36-61) | 38 (33-50) | 0.10 |
| 6 years | 47 (35-63) | 46 (25-49) | 0.70 |
| 7 years | 47 (34-65) | 40 (23-48) | 0.30 |

Data are presented as median (interquartile range). eGFR, estimated glomerular filtration rate.
